# In-Hospital Mortality of Disseminated Tuberculosis in Patients Infected with the Human Immunodeficiency Virus

**DOI:** 10.1155/2011/120278

**Published:** 2010-08-04

**Authors:** Rodrigo Pires dos Santos, Caroline Deutschendorf, Karin Scheid, Luciano Zubaran Goldani

**Affiliations:** ^1^Infectious Disease Service, Hospital de Clínicas de Porto Alegre (HCPA), Universidade Federal do Rio Grande do Sul (UFRGS), 90035-003, Porto Alegre, RS, Brazil; ^2^Comissão de Controle de Infecção, Hospital de Clínicas de Porto Alegre (HCPA), 90035-003 Ramiro Barcelos 2350, Porto Alegre, RS, Brazil

## Abstract

*Background*. Tuberculosis (TB) is a cause of significant morbidity and mortality in patients with AIDS. The goal of our study was to determine predictors of in-hospital mortality in patients with AIDS and disseminated tuberculosis in a middle-income country. *Material and Methods*. We conducted a retrospective cohort study in a tertiary care center, for patients with AIDS in southern Brazil. From 1996 to 2008, all patients with the diagnosis of disseminated TB were included. *Results*. Eighty patients were included. In-hospital mortality was 35%  (*N* = 28). On multivariate Cox regression analysis, low basal albumin (*P* < .01) was associated with death, and fever at admission was related to better survival (*P* < .01). *Conclusion*. Albumin levels or fever are independent predictors of survival in patients with HIV and disseminated TB. They can serve as indirect markers of immunodeficiency in patients with disseminated TB and AIDS.

## 1. Introduction

The advent of highly active antiretroviral therapy (HAART) has changed the natural history of most opportunistic infections and reduced mortality rates in patients with AIDS [1, 2]. For *Mycobacteria tuberculosis* (MTB) disease, several studies have demonstrated the protective effect of HAART, in HIV-infected patients [3–6]. However, tuberculosis (TB) is still a potentially fatal complication in these patients [7, 8]. 

Several risk factors are associated with elevated risk of death in patients with TB: advanced age, drug resistance, delayed specific therapy, lack of rifampicin use, inadequate therapy, and HIV infection [9–11]. Few studies addressed this risk in patients with HIV and disseminated TB [12, 13]. The goal of our study was to determine predictors of in-hospital mortality in patients with AIDS and strictly disseminated TB. 

## 2. Material and Methods

### 2.1. Study Population

We performed a retrospective cohort study at Hospital de Clínicas de Porto Alegre, a 740-bed tertiary referral hospital for persons with AIDS in southern Brazil. From 1996 to 2008, all persons who attended as inpatient and had the diagnosis of HIV infection and disseminated TB were included. Patients were selected from the review of microbiology laboratory records, and data were collected by the review of patient's charts. The study was approved by the ethics committee of Hospital de Clínicas de Porto Alegre. 

### 2.2. Definitions

Disseminated tuberculosis was defined as identification of MTB in blood, or bone marrow, or liver biopsy cultures, as suggested by Wang et al. [14]. Only culture positive subjects were included.

The diagnosis of HIV was made according to current recommendations. For isolation of mycobacteria [15, 16], a radiometric system (BACTEC 460 TB—Becton & Dickinson Diagnostic Instrument Systems, Sparks, MD, USA), and nonradiometric systems (BACTEC 9240—Becton & Dickinson Diagnostic Instrument Systems, Sparks, MD, USA; and, BacT/Alert 240—bioMérieux, Marcy-Etoile, France) were used. For identification of MTB, *NAP* (*p-nitro-alfa-acetylamino-beta-hydroxypropiophenone*) test was performed. This test has specificity of 99% of the identification of *Mycobacterium tuberculosis* complex [17]. In cases which *NAP* test could not be done (*N* = 19, 23%), the identification of species was made by the visualization of the aspect of the colony—presence or absence of cord formation—due to the high sensitivity and specificity of this method [18, 19].

Demographic, clinical, laboratorial, pharmacy, and survival data were collected by chart review. Fever was defined as an axilar temperature ≥38.0°C. Information on concomitant AIDS-related opportunistic infections was collected according to CDC lists [20].

Laboratory data were included, if the sample was collected during the first seven days of hospitalization. CD4 cell count and viral loads were included if they had been determined within six months of hospitalization. Antiretroviral therapy was considered the use of one or more drugs with activity against HIV. Highly active antiretroviral therapy was considered the use of a combination of three or more antiretroviral drugs for HIV therapy. Adequate antituberculous therapy was the use of at least three active drugs against TB for at least seven days, at any time during hospitalization.

### 2.3. Statistical Analysis

A descriptive analysis of the variables collected from each patient was performed. Continuous variables are shown as median values and the dispersion measurement as range values. The chi-squared test or Fisher's exact test were used for univariate analysis of selected categorical variables. Associations were considered statistically significant when *P* value was ≤.05. Multivariate hazard ratios (HR), along with 95% confidence intervals (CI), were calculated using the Cox proportional hazards regression model. We included in the final model, the variables with *P*-value ≤.05 by univariate analysis. Survival analysis was based on Kaplan-Meier method and comparisons of survival curves were made by the log-rank test. Data were analyzed in SPSS 16.0 version program. 

## 3. Results

Eighty-two patients with disseminated tuberculosis were identified. Two patients (1.5%) were excluded from the analysis for lack of data. All the patients had clinical or immunological diagnosis of AIDS. The characteristics of patients are shown in Table 1. Forty-nine patients (61%) were identified by positive blood cultures, twenty-two (27.5%) by bone marrow culture, two patients by liver biopsies culture (2.5%), five patients (6.5%) by blood culture and bone marrow culture, and another two patients (2.5%) by a combination of liver biopsy and bone marrow culture. The median duration of hospitalization was 24 days. 

Eighty-six (*N* = 69) percent of the patients had an abnormal X-ray at admission. Sixty-one patients had sputum analysis. Of these, 32.5% (*N* = 26) were positive for acid-fast bacilli. 

Fifty-five isolates (68.5%) were tested for resistance to antituberculous drugs. One isolate was resistant to rifampicin and pirazinamide, other was resistant to isoniazid and streptomycin, and four isolates were resistant to streptomycin. None of the isolates had multidrug-resistant tuberculosis (i.e., resistance to isoniazid and rifampicin). Any drug resistance was not associated with increased patient mortality (*P* = .67). Rifampicin, isoniazid and pirazinamide, was the initial therapy in 83.5% of patients (*N* = 67). The median time to therapy was seven days.

In-hospital mortality was 35% (*N* = 28). Table 2 shows the univariate analysis related to in-hospital death. Low median albumin levels, low hematocrit and hemoglobin levels, high bilirrubin levels, and ICU admission were all related to in-hospital death on univariate analysis. Fever at admission and appropriate initial TB therapy were related to better survival. Considering other clinical presentations—respiratory symptoms (dyspnea, cough, chest pain), gastrintestinal symptoms (nausea, vomiting, abdominal pain, diarrhea), adenopathies, weight loss, night sweats, headache or skin lesions—they were not related to death on univariate analysis. Eighteen patients (33.5%) diagnosed by blood culture died, compared to 10 patients (38.5%) without mycobacteremia (*P* = .80).

The median time to initiating therapy in patients with fever was 8 days comparing to four days in patients without fever at admission (*P* = .27). The median CD4 cell count was 39 cells/mcL in patients with fever, and 45 cells/mcL in patients without fever at admission (*P* = .57). Median levels of albumin in patients with fever were 2.6 g/dL, and 2.3 g/dL in patients without fever at admission (*P* = .21).

As shown in Table 3, the variables included in Cox regression analysis were those with statistical significance on univariate analysis, and concomitant opportunistic infections because of their clinical relation to death. By multivariate analysis, high albumin levels (HR 0.16, CI 0.05–0.56; *P* < .01) and fever at admission (HR 0.18, CI 0.06–0.54; *P* < .01) were independently related to better survival. Appropriate initial TB therapy was marginally related to better outcomes (HR 0.25, CI 0.06–1.06; *P* = .06). Including CD4 counts in the analysis, high albumin levels (HR 0.25, CI 0.07–0.9; *P* = .04) and fever at admission (HR 0.20, CI 0.05–0.9; *P* = .03) persisted as independent predictors of survival.

**Figure 1:**
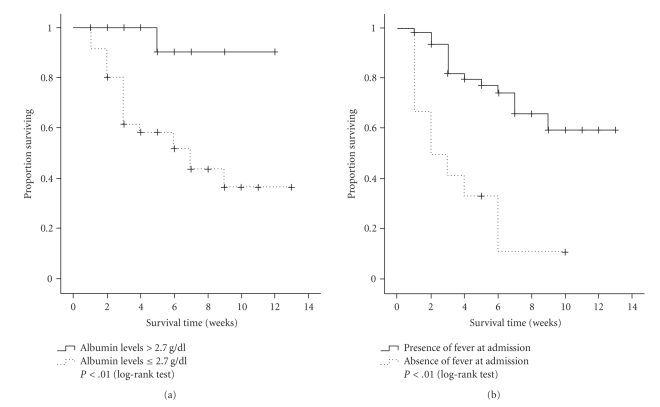
The Kaplan-Meier survival estimates of HIV-infected patients with disseminated TB (a) in relation to basal albumin levels and (b) presence of fever at admission.

Kaplan-Meier curves, as shown in Figure 1, included patients with TB stratified by albumin levels (≤2.7 g/dL versus >2.7 g/dL) at hospital admission and presence or absence of fever at admission. Patients with levels of albumin ≤2.7 g/dL had reduced survival rates (*P* < .01; log-rank test). The patients with fever at hospital admission had significant better survival rates (*P* < .01; log-rank test).

## 4. Discussion

Disseminated TB is a predictor of worse prognosis for patients with HIV infection [13, 21]. In our cohort, we identified albumin levels and fever at admission as additional independent predictors of mortality. These predictors were more strongly associated with mortality than recent CD4 count.

In-hospital mortality in our patients was 35%, similar to prior studies in Thailand and Malawi [22]. Several studies have addressed predictors of mortality in patients with TB. Risk factors for mortality include older age, presence of other comorbid illnesses, emergency department admissions [23]; corticosteroid use [24]; presence of seizures or coma in patients with tuberculous meningitis [25]; multiple organ failure and consolidation on chest radiographs, tuberculous-destroyed lungs, Acute Physiology and Chronic Health Evaluation II scores ≥20, and sepsis in patients who suffered acute respiratory failure [26, 27]; acute renal failure, need for mechanical ventilation, chronic pancreatitis, sepsis, acute respiratory distress syndrome, and nosocomial pneumonia in patients admitted to the ICU [28]; None of these studies addressed specifically risk factors for death in patients with disseminated tuberculosis.

Grinsztejn et al. reported that the in-hospital mortality was higher among patients with mycobacterial bacteremia compared to patients without bacteremia, in their study of 42 patients with M. avium and M. tuberculosis infection [12]. In our study, the patients had a priori the diagnosis of disseminated disease. Mycobacteremia per se was not related to worse prognosis when compared to patients without MTB bacteremia. 

Low levels of albumin (≤2.7 mg/dL) during the first seven days in the hospital were an independent predictor of in-hospital death in our patients with disseminated TB. In the EuroSida risk score study and in the study by Sacks et al., body mass index has been implicated with disease progression and death [29, 30]. Matos and Moreira Lemos reported that serum albumin levels were associated with in-hospital death in a 373-person Brazilian TB cohort with 9% HIV coinfection and without disseminated TB disease [10]. Another study in Israel [25] found that cachexia and lower albumin levels were related to mortality on multivariate analysis. In the Israeli study, 49 (11%) patients were infected with HIV. Furthermore, hypoalbuminaemia is a marker of severe malnutrition and was a predictor of low CD4 counts in HIV-negative TB patients [31]. 

Several cytokines have the capacity to induce fever and play an important role in the modulation of the immune response. The list of pyrogenic cytokines includes, among others, interleukin (IL)–1, (TNF)-alpha, IL-6, and IFN-gamma [32]. Secretion of these proinflammatory cytokines results in fever and the general constitutional symptoms associated with MTB as a result of the active adaptive cell-mediated immune response to MTB. This adaptive response is lead by IFN-gamma secreted by antigen specific T-helper Type 1 (Th1) cells. Several studies have demonstrated that IFN-gamma levels in MTB patients correlate with fever [33, 34]. Thus, the presence of fever likely represents some capacity to mount a partial immune response to MTB, despite the advanced immunosuppression. Hiraki et al. showed that the pleural fluid concentrations of IL-12, IL-18, and IFN-gamma in tuberculosis patients were correlated to high fever (>38°C) [35].

Patients with fever at admission had better in-hospital outcomes in our study. Our patients were severely immunossuppressed (median CD4 39.5 cells/mcL), but only 44 patients had CD4 T cells measured. Although fever was not related to CD4 cell count in our sample and we did not evaluate levels of cytokines and IFN-gamma, the presence of fever at admission could indicate the patients with better immune response against MTB and, consequently, predict those with better outcomes.

Considering that most patients (91.5%) were adequately treated following standard guidelines, the short median time to initiate therapy after the diagnosis, the good in-hospital compliance to anti-TB medication, and the low level of drug resistance (3.5%) to first-line tuberculostatic agents, we can state that our patients seemed to have an aggressive form of disease and/or were diagnosed in their late stage of disseminated tuberculosis. 

Our study has the limitations related to a single-site retrospective cohort study. Firstly, the selection bias: we included only patients with positive cultures for TB, possibly excluding patients with less severe disease, in which blood cultures were less likely to be obtained. Moreover, the missing data, related to the retrospective review, reduced the sample size in the multivariate analysis, which could result in overfitting problem (high number of variables included in relation to events) in the Cox regression analysis. Finally, the observational design precludes us to make definite conclusions about specific TB therapy and use of HAART in relation to outcome.

## 5. Conclusions

Disseminated TB was associated with a high in-hospital mortality rate, despite adequate therapy, state-of-the-art diagnostics, and clinical care. For this reason, we emphasize the importance of HIV testing, access to antiretroviral therapy, and screening and treatment of latent tuberculosis infection in order to prevent the disease and mortality in patients with AIDS. Low basal albumin levels were independently related to in-hospital mortality, and fever at admission was related to better prognosis in HIV-infected patients with disseminated TB and AIDS.

## Figures and Tables

**Table 1:** Characteristics of the HIV-infected patients with disseminated tuberculosis.

Median age (range)	33.0 (14–73)
Men (%)	59 (69.5)
Race	
White (%)	52 (66)
Black (%)	22 (28)
Other (%)	5 (6)
HIV exposure	
Sexual (%)	31 (52.5)
IV drug use (%)	28 (48.5)
Positive Tuberculin Skin Test	2 (5.5)
Previous ART^1^ (%)	20 (25)
Concomitant opportunistic infection^2^ (%)	12 (15)
Median length of hospitalization in days (range)	24 (4–367)
Median CD4 in cells/mcL (No. of patients)	39.5 (44)
Median viral load in log/mL (No. of patients)	4.7 (5)
Anti-HCV positive (%)	24 (39.5)
HBsAg positive (%)	6 (10)
Appropriate initial TB therapy (%)	73 (91)
Median time to TB therapy in days (range)	7 (0–53)
Median time to death in days (range)	18.5 (2–86)

Note. MTB, *Mycobacterium tuberculosis; *IV, intravenous; ART, antiretroviral therapy.

^1^Previous use of ART means previous history of ART.

^2^Opportunistic diseases were as follows: cytomegalovirus infection (*N* = 3), *Cryptococcus neoformans* infection (*N* = 8), esophageal candidiasis (*N* = 6), toxoplasmosis (*N* = 3), pneumocystis pneumonia (*N* = 4), *Cryptosporidium* spp (*N* = 2).

**Table 2:** In-hospital mortality among HIV-infected patients with disseminated tuberculosis.

	In-hospital death			
	Yes	No	Total	*P*
Male	22 (78.5)	37 (71)	59 (74)	.60
Median Age in years (range)	33 (16–73)	33 (14–70)	33 (*N* = 78)	.75
Concomitant opportunistic infection	3 (10.5)	9 (17.5)	12 (15)	.53
ICU admission	14 (50)	4 (7.5)	18 (22.5)	<.01
Fever at admission	17 (60.5)	50 (96)	67 (84)	<.01
Median alkaline phosphatase (U/L)	601	401	426 (*N* = 71)	.58
(range)	(62–2539)	(50–4991)		
Median ALT (U/L)	47	43	43 (*N* = 74)	.41
(range)	(10–389)	(9–429)		
Median AST (U/L)	100	58	69 (*N* = 76)	.82
(range)	(18–544)	(12–1575)		
Median LDH (U/L)	730	723	725 (*N* = 71)	.91
(range)	(227–1627)	(172–2329)		
Median Bilirrubin (mg/dL)	1.3	0.7	0.8 (*N* = 72)	.02
(range)	(0.3–8.1)	(0.3–5.2)		
Median Hematocrit (%)	23	27	26 (*N* = 80)	.03
(range)	(13–34)	(8–43)		
Median Hemoglobin (g/dL)	7.6	8.9	8.8 (*N* = 80)	.02
(range)	(5.5–11.1)	(4.6–13.4)		
Median Leukocytes (10^9^/L)	4.4	5.1	4.8 (*N* = 80)	.14
(range)	(0.5–10.2)	(0.5–31.6)		
Median Albumin (g/dL)	2.2	2.7	2.6 (*N* = 67)	<.01
(range)	(1.4–3.1)	(1.7–4.0)		
Median Creatinine (mg/dL)	0.9	0.8	0.9 (*N* = 79)	.16
(range)	(0.2–2.9)	(0.4–1.7)		
Median CD4 (cells/mcL)	36	39	39 (*N* = 44)	.92
(range)	(1–327)	(2–469)		
In-hospital HAART^1^	6 (22.2)	7 (14.3)	13 (17.1)	.53
Appropriate initial TB therapy	23 (82.1)	50 (96.2)	73 (91.2)	.04
Median time to therapy (days)	6.5	7.0	7.0 (*N* = 70)	.54
(range)	(0.0–49.0)	(0.0–53.0)		

Note. Data are no. (%) of patients, unless otherwise indicated. ICU, Intensive care unit; AFB, acid fast bacilli; ALT, alanine aminotransferase; AST, aspartate aminotransferase; LDH, lactate dehydrogenase; HAART, highly active antiretroviral therapy; TB, tuberculosis.

^1^In-hospital HAART means the prescription of HAART during hospital stay, independent of previous ART use.

**Table 3:** Cox regression analysis. Predictors of in-hospital mortality in HIV-infected patients with disseminated tuberculosis (*N* = 59).

	Hazard Ratio (HR)	Confidence Interval	*P*
Fever at admission	0.18	0.06–0.54	<.01
High albumin levels	0.16	0.05–0.56	<.01
Appropriate initial TB therapy	0.25	0.06–1.06	.06
High bilirrubin levels	1.09	0.88–1.35	.41
Concomitant opportunistic infection	0.52	0.09–3.06	.47
Low hemoglobin levels	1.06	0.82–1.36	.64
ICU admission	0.98	0.33–2.57	.97

Note. HR, hazard ratio for hospital death; ICU, intensive care unit; TB, tuberculosis. High or low levels were per 1 g/dL change for albumin and hemoglobin and per 1 mg/dL for bilirrubins.
